# Is *Cryptococcus neoformans* a pleomorphic fungus?

**DOI:** 10.1016/j.mib.2024.102539

**Published:** 2024-09-10

**Authors:** Jessica CS Brown, Elizabeth R Ballou

**Affiliations:** 1 School of Biological Sciences, University of Utah, Salt Lake City, UT 84112, USA; 2 Medical Research Council Centre for Medical Mycology at the University of Exeter, Exeter EX4 4QD, UK

## Abstract

Improved understanding of the human fungal pathogen *Cryptococcus neoformans*, classically described as a basidiomycete budding yeast, has revealed new infection-relevant single cell morphologies *in vivo* and *in vitro*. Here, we ask whether these morphologies constitute true morphotypes, requiring updated classification of *C. neoformans* as a pleomorphic fungus. We profile recent discoveries of *C. neoformans* seed cells and titan cells and provide a framework for determining whether these and other recently described single-cell morphologies constitute true morphotypes. We demonstrate that multiple *C. neoformans* single-cell morphologies are transcriptionally distinct, stable, heritable, and associated with active growth and therefore should be considered true morphotypes in line with the classification in other well-studied fungi. We conclude that *C. neoformans* is a pleomorphic fungus with an important capacity for morphotype switching that underpins pathogenesis.

## Introduction

The fungal kingdom is diverse and beautiful, encompassing everything from macrostructures (mushroom fruiting bodies) to microscopic single-celled organisms. Changes in morphotype are a common strategy employed by human fungal pathogens, which tend to be single celled, upon infection. The temperature-dependent dimorphic switch from hyphal to yeast form, for example, enables immune evasion and dissemination [1]. For *Cryptococcus* species, *Candida glabrata*, and other budding yeasts that lack a stable infectious hyphal morphotype, the budding yeast form has historically been considered the sole morphotype. However, the term ‘budding yeast’ is inexact, and recent work suggests that this form contains many more morphological variants and stable subpopulations than previously thought. For example, different variants within the budding yeast population of a single species can be transcriptionally distinct and self-propagating [2–4]. Here, we argue that, for *Cryptococcus neoformans*, these variants represent distinct subpopulations worthy of the ‘morphotype’ designation. We conclude that impressive variation in morphology exists and that this directly underpins pathogenesis.

**Figure 1:**
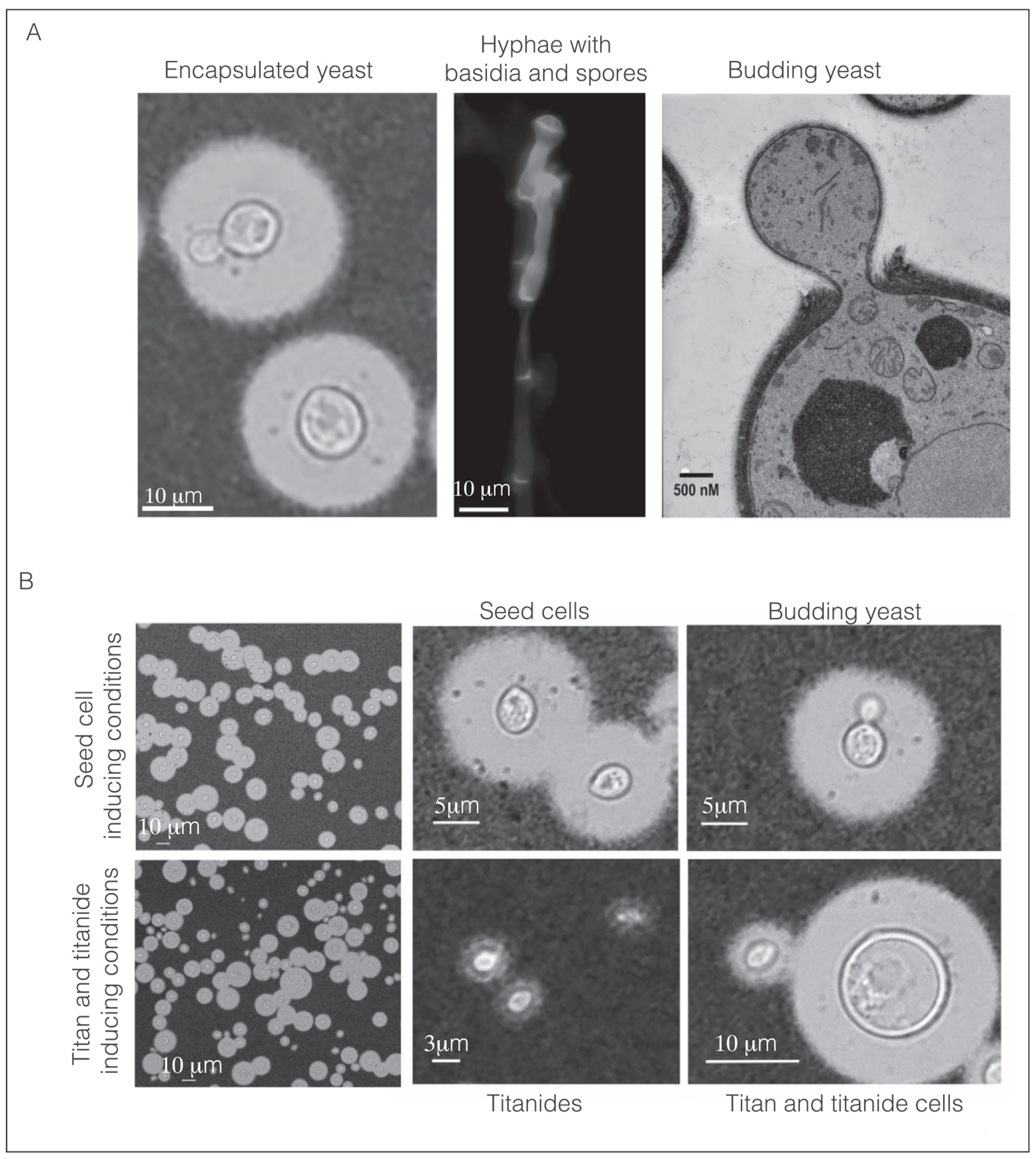
Cryptococcus Morphotypes. **(a)** Recognized Cryptococcus morphotypes: (left) Yeast-phase cells are identified as *Cryptococcus* through the production of a robust capsule revealed by india ink. (Middle) Hypha with clamp cells terminate in two basidia, with spores visible emerging from the top. Hyphae resulting from KN99 MatA x KN99 MatAlpha mating on Murashige-Skoog medium for 7 days were stained with calcofluor white for chitin. (Right) A budding yeast imaged by TEM reveals the layers of cell wall resulting from progressive rounds of budding. **(b)** Newly identified morphotypes: (top) seed cell culture (Denham et al., 2022) generates encapsulated seed cells (4–6 μm), comparable in size and shape to budding yeast (4–6 μm). (bottom) Titan culture (Dambuza et al., 2018) generates a heterogenous population, including titanides (2–3 μm oval cells with minimal capsule) and titan cells (> 10 μm). TEM, Transmission Electron Microscopy.

The human fungal pathogen *Cryptococcus neoformans* is readily identified in patient samples by its distinctive and unique morphology: a budding yeast surrounded by a dramatic polysaccharide capsule that easily distinguishes it from other fungi (Figure 1a). However, this classical presentation belies a complex and fascinating biology that we are only beginning to fully appreciate. As a basidiomycete, Cryptococcus yeast-phase growth is distinct in key ways from ascomycete budding yeasts, including cell wall organization, molecular regulation of polarity, bud site positioning and emergence, nuclear dynamics and cellular division, and genome organization [5–11]. As sexual dimorphs, *Cryptococcus* spp. can switch to filamentous growth in the context of nitrogen limitation or temperature (monokaryotic fruiting) or upon cell–cell fusion (dikaryotic hyphae), which terminate in basidia that produce dispersible basidiospore (Figure 1a) thought to be important for infection of the mammalian lung [12–14].

In the context of host infection, *Cryptococcus* haploid cells can undergo a variety of morphological transitions 15]. The majority of cells during infection are encapsulated haploid yeast, approximately 5–7 μm in size [16,17]. Very rarely, there have been reports of pseudohyphae during infection [18–22]. Most dramatically, yeast can engage an inducible change in body plan to become large, polyploid titan cells that propagate by budding, and these are observed both *in vivo* and *in vitro* in response to a variety of signals [23–30]. Yet small cells are also relevant: The production of very small (1 μm), round, thick-walled ‘microcells’ has been documented *in vivo* and in clinical isolates [31,32]. Thin-walled oval ‘titanides’, 2–3 μm, have been observed *in vitro* and *in vivo* [27,33]. Finally, infection-relevant ‘seed cells’, 4–6 μm, comparable in size to *in vitro* grown yeast, were recently identified *in vivo* as being critical for dissemination, and these are linked to growth in phosphate-rich bird guano, a major environmental niche [17]. Together, these diverse, environmentally relevant morphologies suggest *Cryptococcus* is in fact pleomorphic and that these morphological changes underpin environmental stress resistance and pathogenesis. In this review, we lay out the evidence for and against such an expanded understanding of *C. neoformans* cell biology.

## How do we define morphotypes?

In the dimorphic fungi, yeast and hyphae both meet several important criteria that establish them as ‘forms’ and not just variants within a population of high phenotypic divergence. (1) Each morphotype has a transcriptional signature [34]. (2) Each morphotype is either directly reproduced in the daughter or the mother dictates a distinct morphotype of the daughter. The daughter then replicates faithfully in the new form. This ability is therefore passed down across generations, rather than being a transient, phenotypic response to a stimulus. (3) Morphotypes are distinct from terminal sexual structures, which have distinct, developmentally relevant morphologies and definitions.

The three criteria are easily met for the yeast and hyphae forms of thermal dimorphs, such as *Histoplasma capsulatum* and *Blastomyces dermatitidis* [1]. *Coccidioides* species, while classified as dimorphs, also have multiple, nonsexual hyphae and spherule morphotypes [35]. *Candida* species are often dimorphic, with both yeast and hyphal phases, and recent work has identified multiple morphotypes within the yeast phase of *Candida albicans* [36]. The classic example is white versus opaque cells, which are transcriptionally and phenotypically distinct and reasonably stable due to self-propagating transcriptional networks [37]. Two recently discovered cell states, gray [3] and gastrointestinal-induced transition cells [4,38], can occur during infection [39]. *C. albicans* also produce ‘Goliath cells’, which exhibit distinct adhesive and stress resistance phenotypes [40]. This diversity of morphotypes, beyond sexual and thermal dimorphism, has led to the concept of *Candida albicans* as a pleomorphic fungus.

Applying these definitions, we can distinguish single-cell cryptococcal morphotypes (Figure 1) from changes that occur over less than one generation, such as capsule and melanin production [41,42], or cell wall changes that occur in response to stress or aging [5,43,44]. We additionally recognize single-cell morphotypes as distinct from morphogenesis during the mating phase (Figure 1a), which involves cell types that cannot directly propagate (i.e. the zygote must polarize to form a hypha and spores must germinate to yeast) [45,46]. Please note that we do not intend to offer proscriptive functional definitions of these morphotypes, as complete descriptions can lag initial discovery. Instead, we hope to highlight the existence of new, distinct single-cell morphotypes with the view that these may have different potentials for proliferation in the environment or for mediating pathogenesis.

## Morphotypes versus developmental states

The existence of *Cryptococcus* mating hyphae have classically not been sufficient to classify *Cryptococcus* species as di- or pleomorphic [1]. Instead, hyphae are a developmental state associated with meiotic processes (the so-called ‘perfect state’) (Figure 1a). In the case of dikaryotic mating, following a/alpha cell fusion, the transient dikaryotic zygote fully polarizes to form septate hyphae. Clamp cells mediate independent mitosis of the a and alpha nuclei and subsequent translocation of parent-derived nuclei to the basidium, where they fuse and undergo meiosis to produce spores [47]. Morphogenesis and progression to meiosis are tightly co-ordinated, with the different developmental forms dictated by cell cycle events, including heterokaryon dynamics (clamp cells), nuclear fusion (basidium), and meiosis (spore emergence) [13,48] (reviewed in Ref. [49]).

Spores and conidia, the asexual propagule of filamentous fungi, are important infectious propagules that fulfill many of the phenotypic requirements of a morphotype: they are transcriptionally distinct and their gross morphology is unique [12,50–54]. However, to propagate they must germinate, entering a transcriptionally distinct state, and form either yeast cells, in the case of *Cryptococcus*, or hyphae, in the case of molds [12,55]. Spores and conidia are therefore transcriptionally distinct but not stable. By our definition, we consider them developmental structures rather than morphotypes.

Cryptococcus species can also undergo monokaryotic fruiting, primarily studied in the model species *C. deneoformans* [56,57]. *C. deneoformans* can undergo same sex mating [58], and there is some evidence that this also occurs in *C. neoformans* environmental isolates [59]. *C*. *deneoformans* isolates can also switch to monokaryotic filaments in response to a temperature-induced G2 arrest [14]. The terminal basidium of these cells is a site of meiosis and genome reduction, and so we consider these to be developmental structures.

## Does *Cryptococcus* exhibit multiple morphotypes?

Given these definitions, we argue that *C. neoformans* has at least three budding cellular states that meet our definition of morphotypes — yeast, seed cells, and titan cells — and could well have several more, including the morphologically distinct titanide and microcells, as well as others relevant to pathogenesis.

## Yeast cells

Yeast phase cells are the most commonly studied form of *C. neoformans* and have historically been considered phenotypically homogenous. However, a growing body of evidence demonstrates that yeast cells are far more varied than previously thought, especially when grown in environments that differ from rich yeast media.

Yeast cells grown in standard lab media are uniformly round, 5–7 μm, haploid, and produce clonal daughters through repeated budding. Importantly, *C. neoformans* are molecularly and phenotypically distinct from other well-studied budding yeasts. The *C. neoformans* cell wall is composed of chitin as well as deacetylated chitosan, along with beta-glucan and mannan, typically 200 nm thick [5,27,60,61]. Unlike *S*. *cerevisiae* and *C. albicans*, which place new daughter cells adjacent to the mother, generating a series of bud scars as cells age [44], *C. neoformans* (and other basidiomycetes) repeatedly bud from the same site, causing a thickening of the cell wall at the bud scar [9] (Figure 1a). This phenotypic difference is underpinned by molecular rewiring of polarity compared to *S*. *cerevisiae* [6–8,10,11]. *C. neoformans* has a well-defined cell cycle, yet the transcriptional programs that regulate transitions between cell cycle phases are distinct from those of *S*. *cerevisiae* [62]. Moreover, nuclear dynamics during mitosis are unique, with the nucleus moving into the daughter cell and dividing across the mother/daughter neck via semiopen mitosis [10]. Under host-relevant conditions, Cryptococcus cells produce melanin and an elaborate GXM/GalXM capsule [41,63] and also secrete factors associated with virulence, including urease and the cysteine-rich secreted factor Cpl1 [64,65]. Finally, specific transcriptional programs mounted in response to stress and environmental conditions in ascomycetes [66,67] underpin pathogenesis in *Cryptococcus* [68–71].

## Seed cells

Seed cells (Figure 1b) are a smaller (~5 μm cell body, ~7 μm cell body + capsule) morphotype that appear in the lungs and brain around the time that *C*. *neoformans* cells start to disseminate in extrapulmonary organs [17] and correlate with brain fungal burden across different strains and species of *Cryptococcus*. Compared with encapsulated or yeast medium-grown unencapsulated yeast, seed cells have different cell surfaces, including increased conconavalin A binding, which suggests increased exposure of mannoproteins or other mannose moieties. This mannose exposure results in greater fungal cell uptake into the liver. Seed cells are also better able to enter the brain compared with encapsulated or yeast medium-grown cells. They are inducible and have statistically distinct transcriptomes from encapsulated yeast cells, identifiable by principle component analysis, and appear to have haploid genomes. *In vitro*, phosphate is sufficient to cause encapsulated yeast cells to produce seed cell daughters (Figure 1b, and personal communication, Jessica Brown). Pigeon guano, one of the environmental niches of *C. neoformans* cells, is also sufficient to induce seed cell formation [17]. While much about the specific biology remains to be discovered, seed cells fulfill our definition of a morphotype: transcriptionally distinct, stable with induction, and associated with active growth rather than a mating or developmental phase.

## Titan cells

Titan cells (Figure 1b) are large (> 10 μm cell body, > 15 μm including capsule) *in vivo*–relevant cells that were first described in patient samples [23,24]. These were originally considered a ‘dead-end’ or ‘stress-induced’ host-specific morphology; however, early work demonstrated they can be reproducibly induced *in vivo* and propagate asymmetrically [25,26,31]. Specifically, Nielsen and Zaragoza defined these as cells with a diameter greater than 10 μm, ploidy greater than or equal to 4C, a more densely crosslinked capsule, and a thickened cell wall [27,72]. Work by Mukaramera et al. provided refined understanding of the cell wall composition, highlighting altered chitin/chitosan ratios in large versus small cells recovered from the host lung [73]. Subsequent work by Probert et al. identified differences in capsule epitope localisation during *in vitro* culture [74]. Okagaki et al. also identified that titan cells are associated with reduced phagocytosis of both small and large cells, consistent with the production of a titan cell–associated secreted factor with inhibitory activity against phagocytes [75].

Titan cells emerge when yeast cells are incubated at low density in the presence of specific environmental triggers, including the host environment, nutrient limitation, bacteria, activators of cAMP signaling, and mitochondrial stress [25–30]. However, under *in vitro* and *in vivo* conditions, titan cells comprise a minority of the total cell population (Figure 1b), complicating transcriptional characterisation. Despite this, RNAseq of heterogenous titan cultures suggest they have distinct transcriptional profiles relative to yeast-phase growth, and there are clear indicators of both positive and negative regulation of the morphological switch [28]. CO_2_ is a major activator of cAMP signaling [76], and loss of cAMP signaling inhibits titan cell formation [27,28,77]. In addition, the CO_2_-responsive transcription factor Usv101 negatively regulates titan cell formation [27,78]. Extracellular pH is a key determinant of whether Cryptococcus proliferates as yeast or switches morphotype [17,79], and the pH-responsive transcription factor Rim101 is required for titan cell formation and cell wall integrity [77,80]. High cell density represses titan cell formation [27]. The transcription factor Pdr802 regulates quorum sensing components *OPT1*, *PQP1*, and *LIV3*. and *pdr802*Δ mutants are hypertitanizing, consistent with a failure to sense cell density [81]. However, the exact nature of the quorum sensing molecule that mediates this remains unclear: The addition of exogenous synthetic quorum sensing peptide Qsp1 reduces, but does not completely block, the formation of titan cells, and deletion of *CQS1*, the gene encoding the Qsp1 peptide, does not make cells insensitive to high density, suggesting the existence of additional density-dependent regulators [28].

## Do titan cells fit the criteria of a true morphotype?

Overall, this suggests titan cells are an inducible, transcriptionally distinct cell type; however, the identity of titan cells as a true morphotype is more subtle. Further examination of the events driving titan cell formation may suggest they are more analogous to fruiting bodies than they are to yeast.

The capacity to switch to titan growth appears to be specific to the *Cryptococcus* lineage [82]. Titan cells do not divide symmetrically [72], but titan cells morphology is progressive across generations, with daughter characteristics dictated by mother: Haploid yeast cells endoreduplicate to form uninucleate polyploid titan cells, and these then divide asymmetrically to produce haploid, aneuploid, or diploid daughter cells that in turn have the capacity to form titan cells [25,27,83]. Given their capacity for inducible changes in ploidy, it follows that the formation of titan cells is inextricably linked to cell cycle–dependent events. Titan cells initially form when cells fail to progress through G2 arrest, instead endoreduplicating their genomes, either through exogenous DNA damage or through altered cell cycle regulation [13,84]. Loss of the cyclin Cln1, required for exit from G2, results in elevated titan cell formation under inducing conditions [84]. In addition, the negative regulator Usv101 sits downstream of Swi6 (CNAG_01438), a component of the mitotic SBF/MBF regulatory complex that also regulates capsule and melanin [27,41,78]. Genes linked to the meiotic cell cycle are also implicated in titan cell formation. For example, MAT**a** cells in the KN99 lineage produce titan cells at elevated rates (> 20%), and this is dependent on the alpha pheromone receptor Ste3a [26]. The receptor Gpr5 also appears to be required for *in vivo* and *in vitro* titan cell formation [27,77], likely acting via Gpa1 [77]. Gpa1 localises to the plasma membrane in response to phosphorylation by the Cyclin-dependent Kinase-related kinase Crk1, also involved in meiosis [85,86]. Finally, meiotic genes mediate ploidy reduction in response to genotoxic stress [13]. It has been proposed that the capacity to alter ploidy is a driver of diversification of otherwise clonal populations and may underpin drug resistance [83]. Overall, this raises the possibility that titan cells may be a developmental morphotype with implications for disease outcome.

## Additional potential morphotypes and discovery of new morphotypes

Images of *C. neoformans* in human or murine lungs are impressive in their heterogeneity [24,87,88], and this variance could well indicate additional morphotypes. Several promising candidates have been reported but more data are needed to assess whether these are additional true morphotypes. These include a variety of distinct ‘small cells’ (< 4 μm).

### Titanides

Titanides (Figure 1b) appear in conditions that also induce titan cells, yet their origin remains unclear [27]. Distinct from round yeast and seed cells (both 4–6 μm), titanides are distinctly oval in shape, 2–3 μm on their long axis, and have significantly thinner cell walls than yeast [27]. They have been observed in multiple different *in vitro* assay conditions as well as *in vivo* and are produced by a variety of VNI isolates at varying rates [27,33,89,90].

### Microcells

Microcells were originally defined as < 1 μm with thick cell walls [31]. Their round shape, very small size, thick cell wall and robust capsule easily distinguish them from seed and titanide cells [17,27,32]. They appear to be limited to *C. neoformans* lineages and are associated with the increased virulence of this species relative to other *Cryptococcus* species [32].

### Viable but nonculturable and dormant cells

Another potential morphotype observed *in vivo* are viable but nonculturable (VBNC) cells [91]. Although poorly understood, these may be analogous to bacterial persister cells [92], with intact membranes and low metabolic activity [91]. While whole transcriptome data are lacking, VBNCs are transcriptionally distinct from other, higher metabolism cells for limited set of 37 test genes. They also have relatively high calcofluor white staining, indicative of elevated cell wall chitin levels [91]. When isolated from mice, VBNCs can resume growth in the presence of serum [91] or Sabouraud dextrose agar [93]. Whether VBNCs are resistant to antifungal drugs, as bacterial persister cells are resistant to antibacterial agents, has not been established. However, VBNCs exhibit signs of cellular stress responses [93], which can confer stress adaptation that is important for virulence [94]. Moreover, recent work identified increased antifungal resistance in metabolically dormant *Cryptococcus* subpopulations [95]. Overall, we think that VBNCs could well represent a new morphotype, but the challenges of determining viability and whether different groups are working on the same subpopulation of cells suggest that more evidence is needed.

## Conclusions

Based on the established definition for morphotypes, we conclude that so-called ‘yeast’ phases can be complex, comprising multiple stable, independent morphotypes and that Cryptococcus exhibits multiple, initially subtle morphotypes that are biologically important both to fungal proliferation and to pathogenesis. We argue that *Cryptococcus* should therefore be termed ‘pleomorphic’. We additionally highlight that titan cells may extend the current working definition of morphotype by co-opting meiotic processes normally reserved for developmental forms. Finally, a common thread across the morphotypes is changes in cell wall, antigen exposure, and secreted factors that have important implications for host response [17,27,51,60,64,65,73–75,91,96]. Cell wall changes can also occur during aging [44] or as transient responses to stress [5], independent of morphotype. Overall, it is clear that, while these changes alone do not underpin morphotype definitions, investigations of fungal morphotypes should include a robust analysis of the fungal cell wall and secretome.

## Data Availability

No data were used for the research described in the article.

## References

[1] Sil A, Andrianopoulos A: Thermally dimorphic human fungal pathogens — Polyphyletic pathogens with a convergent pathogenicity trait. Cold Spring Harb Perspect Med 2014, 5:a019794. 25384771 10.1101/cshperspect.a019794 PMC4526722

[2] Hernday AD, Lohse MB, Fordyce PM, Nobile CJ, DeRisi JL, Johnson AD: Structure of the transcriptional network controlling white-opaque switching in *Candida albicans*. Mol Microbiol 2013, 90:22–35. 23855748 10.1111/mmi.12329 PMC3888361

[3] Tao L, Du H, Guan G, Dai Y, Nobile CJ, Liang W, : Discovery of a “white-gray-opaque” tristable phenotypic switching system in *Candida albicans*: roles of non-genetic diversity in host adaptation. PLoS Biol 2014, 12:e1001830. 24691005 10.1371/journal.pbio.1001830 PMC3972085

[4] Pande K, Chen C, Noble SM: Passage through the mammalian gut triggers a phenotypic switch that promotes *Candida albicans* commensalism. Nat Genet 2013, 45:1088–1091. 23892606 10.1038/ng.2710 PMC3758371

[5] Mukaremera L: The Cryptococcus wall: a different wall for a unique lifestyle. PLoS Pathog 2023, 19:e1011141. 36821541 10.1371/journal.ppat.1011141 PMC9949634

[6] Waugh MS, Nichols CB, DeCesare CM, Cox GM, Heitman J, Alspaugh JA: Ras1 and Ras2 contribute shared and unique roles in physiology and virulence of *Cryptococcus neoformans*. Microbiology 2002, 148:191–201. 11782511 10.1099/00221287-148-1-191

[7] Ballou ER, Kozubowski L, Nichols CB, Alspaugh JA: Ras1 acts through duplicated Cdc42 and Rac proteins to regulate morphogenesis and pathogenesis in the human fungal pathogen *Cryptococcus neoformans*. PLoS Genet 2013, 9:e1003687. 23950731 10.1371/journal.pgen.1003687 PMC3738472

[8] Nichols CB, Ferreyra J, Ballou ER, Alspaugh JA: Subcellular localization directs signaling specificity of the *Cryptococcus neoformans* Ras1 protein. Eukaryot Cell 2009, 8:181–189. 19098128 10.1128/EC.00351-08 PMC2643607

[9] Moore RT: Chapter 5 — Cytology and ultrastructure of yeasts and yeastlike fungi. In The Yeasts. Edited by Kurtzman CP, Fell JW. Fourth edition, Elsevier; 1998:33–44.

[10] Kozubowski L, Yadav V, Chatterjee G, Sridhar S, Yamaguchi M, Kawamoto S, : Ordered kinetochore assembly in the human-pathogenic basidiomycetous yeast *Cryptococcus neoformans*. MBio 2013, 4:e00614–13. 24085781 10.1128/mBio.00614-13 PMC3791896

[11] Buscaino A: Chromatin-mediated regulation of genome plasticity in human fungal pathogens. Genes 2019, 10:855, 10.3390/genes10110855 31661931 PMC6896017

[12] Velagapudi R, Hsueh Y-P, Geunes-Boyer S, Wright JR, Heitman J: Spores as infectious propagules of *Cryptococcus neoformans*. Infect Immun 2009, 77:4345–4355. 19620339 10.1128/IAI.00542-09 PMC2747963

[13] Zhao Y, Wang Y, Upadhyay S, Xue C, Lin X: Activation of meiotic genes mediates ploidy reduction during cryptococcal infection. Curr Biol 2020, 30:1387–1396 e5. 32109388 10.1016/j.cub.2020.01.081 PMC7228024

[14] Fu J, Morris IR, Wickes BL: The production of monokaryotic hyphae by *Cryptococcus neoformans* can be induced by high temperature arrest of the cell cycle and is independent of same-sex mating. PLoS Pathog 2013, 9:e1003335. 23658522 10.1371/journal.ppat.1003335 PMC3642078

[15] Kronstad JW, Attarian R, Cadieux B, Choi J, D’Souza CA, Griffiths EJ, : Expanding fungal pathogenesis: Cryptococcus breaks out of the opportunistic box. Nat Rev Microbiol 2011, 9:193–203. 21326274 10.1038/nrmicro2522 PMC4698337

[16] Robertson EJ, Najjuka G, Rolfes MA, Akampurira A, Jain N, Anantharanjit J, : *Cryptococcus neoformans* ex vivo capsule size is associated with intracranial pressure and host immune response in HIV-associated cryptococcal meningitis. J Infect Dis 2014, 209:74–82. 23945372 10.1093/infdis/jit435 PMC3864387

[17.••] Denham ST, Brammer B, Chung KY, Wambaugh MA, Bednarek JM, Guo L, : A dissemination-prone morphotype enhances extrapulmonary organ entry by *Cryptococcus neoformans*. Cell Host Microbe 2022, 30:1382–1400 e8. 36099922 10.1016/j.chom.2022.08.017 PMC9588642

[18] Shadomy HJ, Utz JP: Preliminary studies on a hyphaforming mutant of *Cryptococcus neoformans*. Mycologia 1966, 58:383–390. 5941601

[19] Freed ER, Duma RJ, Shadomy HJ, Utz JP: Meningoencephalitis due to hyphae-forming *Cryptococcus neoformans*. Am J Clin Pathol 1971, 55:30–33. 5099781 10.1093/ajcp/55.1.30

[20] Shadomy HJ, Lurie HI: Histopathological observations in experimental cryptococcosis caused by a hypha-producing strain of *Cryptococcus neoformans* (Coward strain) in mice. Sabouraudia 1971, 9:6–9. 4928768 10.1080/00362177185190031

[21] Williamson JD, Silverman JF, Mallak CT, Christie JD: Atypical cytomorphologic appearance of *Cryptococcus neoformans*: a report of five cases. Acta Cytol 1996, 40:363–370. 8629428 10.1159/000333769

[22] Gazzoni AF, Severo CB, Barra MB, Severo LC: Atypical micromorphology and uncommon location of cryptococcosis: a histopathologic study using special histochemical techniques (one case report). Mycopathologia 2009, 167:197–202. 19052915 10.1007/s11046-008-9169-1

[23] Love GL, Boyd GD, Greer DL: Large *Cryptococcus neoformans* isolated from brain abscess. J Clin Microbiol 1985, 22:1068–1070. 3905847 10.1128/jcm.22.6.1068-1070.1985 PMC271885

[24] Cruickshank JG, Cavill R, Jelbert M: *Cryptococcus neoformans* of unusual morphology. Appl Microbiol 1973, 25:309–312. 4121033 10.1128/am.25.2.309-312.1973 PMC380794

[25] Zaragoza O, García-Rodas R, Nosanchuk JD, Cuenca-Estrella M, Rodríguez-Tudela JL, Casadevall A: Fungal cell gigantism during mammalian infection. PLoS Pathog 2010, 6:e1000945. 20585557 10.1371/journal.ppat.1000945 PMC2887474

[26] Okagaki LH, Strain AK, Nielsen JN, Charlier C, Baltes NJ, Chrétien F, : Cryptococcal cell morphology affects host cell interactions and pathogenicity. PLoS Pathog 2010, 6:e1000953. 20585559 10.1371/journal.ppat.1000953 PMC2887476

[27.••] Dambuza IM, Drake T, Chapuis A, Zhou X, Correia J, Taylor-Smith L, : The Cryptococcus neoformans titan cell is an inducible and regulated morphotype underlying pathogenesis. PLoS Pathog 2018, 14:e1006978. 29775474 10.1371/journal.ppat.1006978 PMC5959070

[28.••] Trevijano-Contador N, de Oliveira HC, García-Rodas R, Rossi SA, Llorente I, Zaballos Á, *Cryptococcus neoformans* can form titan-like cells in vitro in response to multiple signals. PLoS Pathog 2018, 14:e1007007. 29775477 10.1371/journal.ppat.1007007 PMC5959073

[29] Hommel B, Mukaremera L, Cordero RJB, Coelho C, Desjardins CA, Sturny-Leclère A, Titan cells formation in *Cryptococcus neoformans* is finely tuned by environmental conditions and modulated by positive and negative genetic regulators. PLoS Pathog 2018, 14:e1006982. 29775480 10.1371/journal.ppat.1006982 PMC5959062

[30] Saidykhan L, Correia J, Romanyuk A, Peacock AFA, Desanti GE, Taylor-Smith L, An in vitro method for inducing titan cells reveals novel features of yeast-to-titan switching in the human fungal pathogen *Cryptococcus gattii*. PLoS Pathog 2022, 18:e1010321. 35969643 10.1371/journal.ppat.1010321 PMC9426920

[31.••] Feldmesser M, Kress Y, Casadevall A: Dynamic changes in the morphology of *Cryptococcus neoformans* during murine pulmonary infection. Microbiology 2001, 147:2355–2365. 11496012 10.1099/00221287-147-8-2355

[32] Fernandes KE, Brockway A, Haverkamp M, Cuomo CA, van Ogtrop F, Perfect JR, Phenotypic variability correlates with clinical outcome in Cryptococcus isolates obtained from Botswanan HIV/AIDS patients. MBio 2018, 9:e02016–18, 10.1128/mBio.02016-18 PMC6199498 30352938

[33] Xie S, Sao R, Braun A, Bottone EJ: Difference in *Cryptococcus neoformans* cellular and capsule size in sequential pulmonary and meningeal infection: a postmortem study. Diagn Microbiol Infect Dis 2012, 73:49–52. 22424901 10.1016/j.diagmicrobio.2012.01.008

[34] Villa S, Hamideh M, Weinstock A, Qasim MN, Hazbun TR, Sellam A, Transcriptional control of hyphal morphogenesis in *Candida albicans*. FEMS Yeast Res 2020, 20:foaa005, 10.1093/femsyr/foaa005 31981355 PMC7000152

[35] Kirkland TN, Fierer J: Coccidioides immitis and posadasii; A review of their biology, genomics, pathogenesis, and host immunity. Virulence 2018, 9:1426–1435. 30179067 10.1080/21505594.2018.1509667 PMC6141143

[36] Kumamoto CA, Gresnigt MS, Hube B: The gut, the bad and the harmless: *Candida albicans* as a commensal and opportunistic pathogen in the intestine. Curr Opin Microbiol 2020, 56:7–15. 32604030 10.1016/j.mib.2020.05.006 PMC7744392

[37] Slutsky B, Staebell M, Anderson J, Risen L, Pfaller M, Soll DR: “White-opaque transition”: a second high-frequency switching system in *Candida albicans*. J Bacteriol 1987, 169:189–197. 3539914 10.1128/jb.169.1.189-197.1987 PMC211752

[38] Witchley JN, Basso P, Brimacombe CA, Abon NV, Noble SM: Recording of DNA-binding events reveals the importance of a repurposed *Candida albicans* regulatory network for gut commensalism. Cell Host Microbe 2021, 29:1002–1013.e9. 33915113 10.1016/j.chom.2021.03.019 PMC8216204

[39] Liang S-H, Anderson MZ, Hirakawa MP, Wang JM, Frazer C, Alaalm LM, Hemizygosity enables a mutational transition governing fungal virulence and commensalism. Cell Host Microbe 2019, 25:418–431 e6.. 30824263 10.1016/j.chom.2019.01.005 PMC6624852

[40] Malavia D, Lehtovirta-Morley LE, Alamir O, Weiß E, Gow NAR, Hube B, Zinc limitation induces a hyper-adherent goliath phenotype in *Candida albicans*. Front Microbiol 2017, 8:2238. 29184547 10.3389/fmicb.2017.02238 PMC5694484

[41] Lee D, Jang E-H, Lee M, Kim S-W, Lee Y, Lee K-T, Unraveling melanin biosynthesis and signaling networks in *Cryptococcus neoformans*. MBio 2019, 10:e02267–19, 10.1128/mBio.02267-19 31575776 PMC6775464

[42] Casadevall A, Coelho C, Cordero RJB, Dragotakes Q, Jung E, Vij R, The capsule of *Cryptococcus neoformans*. Virulence 2019, 10:822–831. 29436899 10.1080/21505594.2018.1431087 PMC6779390

[43] Yu C-H, Chen Y, Desjardins CA, Tenor JL, Toffaletti DL, Giamberardino C, Landscape of gene expression variation of natural isolates of *Cryptococcus neoformans* in response to biologically relevant stresses. Micro Genom 2020, 6:e000319, 10.1099/mgen.0.000319 PMC7067042 31860441

[44] Silva VKA, Bhattacharya S, Oliveira NK, Savitt AG, Zamith-Miranda D, Nosanchuk JD, Replicative aging remodels the cell wall and is associated with increased intracellular trafficking in human pathogenic yeasts. MBio 2021, 13:e0019022. 35164553 10.1128/mbio.00190-22 PMC8844920

[45] Usher J: The mechanisms of mating in pathogenic fungi — a plastic trait. Genes 2019, 10:831, 10.3390/genes10100831 31640207 PMC6826560

[46] Fu C, Sun S, Billmyre RB, Roach KC, Heitman J: Unisexual versus bisexual mating in *Cryptococcus neoformans*: consequences and biological impacts. Fungal Genet Biol 2015, 78:65–75. 25173822 10.1016/j.fgb.2014.08.008 PMC4344436

[47] Kozubowski L, Heitman J: Profiling a killer, the development of *Cryptococcus neoformans*. FEMS Microbiol Rev 2012, 36:78–94 (Available), ⟨https://academic.oup.com/femsre/article-abstract/36/1/78/538248⟩. 21658085 10.1111/j.1574-6976.2011.00286.x PMC3318972

[48] Liu L, He G-J, Chen L, Zheng J, Chen Y, Shen L, Genetic basis for coordination of meiosis and sexual structure maturation in *Cryptococcus neoformans*. Elife 2018, 7:e38683, 10.7554/eLife.38683 30281018 PMC6235564

[49.••] Zhao Y, Lin X: *Cryptococcus neoformans*: sex, morphogenesis, and virulence. Infect Genet Evol 2021, 89:104731. 33497839 10.1016/j.meegid.2021.104731 PMC8092418

[50] Huang M, Hebert AS, Coon JJ, Hull CM: Protein composition of infectious spores reveals novel sexual development and germination factors in Cryptococcus. PLoS Genet 2015, 11:e1005490. 26313153 10.1371/journal.pgen.1005490 PMC4551743

[51] Botts MR, Giles SS, Gates MA, Kozel TR, Hull CM: Isolation and characterization of *Cryptococcus neoformans* spores reveal a critical role for capsule biosynthesis genes in spore biogenesis. Eukaryot Cell 2009, 8:595–605. 19181873 10.1128/EC.00352-08 PMC2669189

[52] Sephton-Clark PCS, Muñoz JF, Ballou ER, Cuomo CA, Voelz K: Pathways of pathogenicity: transcriptional stages of germination in the fatal fungal pathogen *Rhizopus delemar*. mSphere 2018, 3:e00403–18, 10.1128/mSphere.00403-18 30258038 PMC6158513

[53] Wu M-Y, Mead ME, Lee M-K, Neuhaus GF, Adpressa DA, Martien JI, Transcriptomic, protein-DNA interaction, and metabolomic studies of VosA, VelB, and WetA in *Aspergillus nidulans* asexual spores. MBio 2021, 12:E03128–20, 10.1128/mBio.03128-20 33563821 PMC7885118

[54] Son Y-E, Yu J-H, Park H-S: The novel spore-specific regulator SscA controls *Aspergillus conidiogenesis*. MBio 2023, 14:e0184023. 37707170 10.1128/mbio.01840-23 PMC10653911

[55] Stewart JIP, Fava VM, Kerkaert JD, Subramanian AS, Gravelat FN, Lehoux M, Reducing *Aspergillus fumigatus* virulence through targeted dysregulation of the conidiation pathway. MBio 2020, 11:e03202–19, 10.1128/mBio.03202-19 32019801 PMC7002347

[56] Wickes BL, Mayorga ME, Edman U, Edman JC: Dimorphism and haploid fruiting in *Cryptococcus neoformans*: association with the alpha-mating type. Proc Natl Acad Sci USA 1996, 93:7327–7331. 8692992 10.1073/pnas.93.14.7327 PMC38983

[57] Tscharke RL, Lazera M, Chang YC, Wickes BL, Kwon-Chung KJ: Haploid fruiting in *Cryptococcus neoformans* is not mating type α-specific. Fungal Genet Biol 2003, 39:230–237. 12892636 10.1016/s1087-1845(03)00046-x

[58] Lin X, Hull CM, Heitman J: Sexual reproduction between partners of the same mating type in *Cryptococcus neoformans*. Nature 2005, 434:1017–1021. 15846346 10.1038/nature03448

[59] Lin X, Patel S, Litvintseva AP, Floyd A, Mitchell TG, Heitman J: Diploids in the *Cryptococcus neoformans* serotype A population homozygous for the alpha mating type originate via unisexual mating. PLoS Pathog 2009, 5:e1000283. 19180236 10.1371/journal.ppat.1000283 PMC2629120

[60] Wang ZA, Li LX, Doering TL: Unraveling synthesis of the cryptococcal cell wall and capsule. Glycobiology 2018, 28:719–730. 29648596 10.1093/glycob/cwy030 PMC6142866

[61] Garcia-Rubio R, de Oliveira HC, Rivera J, Trevijano-Contador N: The fungal cell wall: Candida, Cryptococcus, and Aspergillus species. Front Microbiol 2019, 10:2993. 31993032 10.3389/fmicb.2019.02993 PMC6962315

[62.••] Kelliher CM, Leman AR, Sierra CS, Haase SB: Investigating conservation of the cell-cycle-regulated transcriptional program in the fungal pathogen, *Cryptococcus neoformans*. PLoS Genet 2016, 12:e1006453. 27918582 10.1371/journal.pgen.1006453 PMC5137879

[63] García-Rodas R, Cordero RJB, Trevijano-Contador N, Janbon G, Moyrand F, Casadevall A, Capsule growth in *Cryptococcus neoformans* is coordinated with cell cycle progression. MBio 2014, 5:e00945–14. 24939886 10.1128/mBio.00945-14 PMC4056547

[64] Dang EV, Lei S, Radkov A, Volk RF, Zaro BW, Madhani HD: Secreted fungal virulence effector triggers allergic inflammation via TLR4. Nature 2022, 608:161–167. 35896747 10.1038/s41586-022-05005-4 PMC9744105

[65] Cox GM, Mukherjee J, Cole GT, Casadevall A, Perfect JR: Urease as a virulence factor in experimental cryptococcosis. Infect Immun 2000, 68:443–448. 10639402 10.1128/iai.68.2.443-448.2000 PMC97161

[66] Gasch AP, Spellman PT, Kao CM, Carmel-Harel O, Eisen MB, Storz G, Genomic expression programs in the response of yeast cells to environmental changes. Mol Biol Cell 2000, 11:4241–4257. 11102521 10.1091/mbc.11.12.4241 PMC15070

[67] Brown AJP, Budge S, Kaloriti D, Tillmann A, Jacobsen MD, Yin Z, Stress adaptation in a pathogenic fungus. J Exp Biol 2014, 217:144–155. 24353214 10.1242/jeb.088930 PMC3867497

[68] Zhao Y, Lin X: A PAS protein directs metabolic reprogramming during cryptococcal adaptation to hypoxia. MBio 2021, 12:e03602–20, 10.1128/mBio.03602-20 33727360 PMC8092316

[69] Upadhya R, Campbell LT, Donlin MJ, Aurora R, Lodge JK: Global transcriptome profile of *Cryptococcus neoformans* during exposure to hydrogen peroxide induced oxidative stress. PLoS One 2013, 8:e55110. 23383070 10.1371/journal.pone.0055110 PMC3557267

[70] O’Meara TR, Xu W, Selvig KM, O’Meara MJ, Mitchell AP, Alspaugh JA: The *Cryptococcus neoformans* Rim101 transcription factor directly regulates genes required for adaptation to the host. Mol Cell Biol 2014, 34:673–684. 24324006 10.1128/MCB.01359-13 PMC3911494

[71] Summers DK, Perry DS, Rao B, Madhani HD: Coordinate genomic association of transcription factors controlled by an imported quorum sensing peptide in *Cryptococcus neoformans*. PLoS Genet 2020, 16:e1008744. 32956370 10.1371/journal.pgen.1008744 PMC7537855

[72] Zaragoza O, Nielsen K: Titan cells in *Cryptococcus neoformans*: cells with a giant impact. Curr Opin Microbiol 2013, 16:409–413. 23588027 10.1016/j.mib.2013.03.006 PMC3723695

[73] Mukaremera L, Lee KK, Wagener J, Wiesner DL, Gow NAR, Nielsen K: Titan cell production in Cryptococcus neoformans reshapes the cell wall and capsule composition during infection. Cell Surf 2018, 1:15–24. 30123851 10.1016/j.tcsw.2017.12.001 PMC6095662

[74] Probert M, Zhou X, Goodall M, Johnston SA, Bielska E, Ballou ER, A glucuronoxylomannan epitope exhibits serotype-specific accessibility and redistributes towards the capsule surface during titanization of the fungal pathogen *Cryptococcus neoformans*. Infect Immun 2019, 87:e00731–18 ⟨https://iai.asm.org/content/87/4/e00731-18.abstract⟩. 30670549 10.1128/IAI.00731-18 PMC6434129

[75] Okagaki LH, Nielsen K: Titan cells confer protection from phagocytosis in *Cryptococcus neoformans* infections. Eukaryot Cell 2012, 11:820–826. 22544904 10.1128/EC.00121-12 PMC3370461

[76] Caza M, Kronstad JW: The cAMP/protein kinase a pathway regulates virulence and adaptation to host conditions in *Cryptococcus neoformans*. Front Cell Infect Microbiol 2019, 9:212. 31275865 10.3389/fcimb.2019.00212 PMC6592070

[77] Okagaki LH, Wang Y, Ballou ER, O’Meara TR, Bahn Y-S, Alspaugh JA, Cryptococcal titan cell formation is regulated by G-protein signaling in response to multiple stimuli. Eukaryot Cell 2011, 10:1306–1316. 21821718 10.1128/EC.05179-11 PMC3187071

[78] Gish SR, Maier EJ, Haynes BC, Santiago-Tirado FH, Srikanta DL, Ma CZ, Computational analysis reveals a key regulator of cryptococcal virulence and determinant of host response. MBio 2016, 7:e00313–16. 27094327 10.1128/mBio.00313-16 PMC4850258

[79] Dyląg M, Colón-Reyes RJ, Loperena-Álvarez Y, Kozubowski L: Establishing minimal conditions sufficient for the development of titan-like cells in *Cryptococcus neoformans/gattii* species complex. Pathogens 2022, 11:768, 10.3390/pathogens11070768 35890013 PMC9322185

[80] O’Meara TR, Holmer SM, Selvig K, Dietrich F, Alspaugh JA: *Cryptococcus neoformans* Rim101 is associated with cell wall remodeling and evasion of the host immune responses. MBio 2013, 4:e00522–12, 10.1128/mbio.00522-12 23322637 PMC3551547

[81] Reuwsaat JCV, Agustinho DP, Motta H, Chang AL, Brown H, Brent MR, The transcription factor Pdr802 Regulates titan cell formation and pathogenicity of *Cryptococcus neoformans*. MBio 2021, 12:e03457–20, 10.1128/mbio.03457-20 33688010 PMC8092302

[82] Dyląg M, Colon-Reyes RJ, Kozubowski L: Titan cell formation is unique to Cryptococcus species complex. Virulence 2020, 11:719–729. 32498590 10.1080/21505594.2020.1772657 PMC7549989

[83] Gerstein AC, Fu MS, Mukaremera L, Li Z, Ormerod KL, Fraser JA, Polyploid titan cells produce haploid and aneuploid progeny to promote stress adaptation. MBio 2015, 6:e01340–15. 26463162 10.1128/mBio.01340-15 PMC4620463

[84] Altamirano S, Li Z, Fu MS, Ding M, Fulton SR, Yoder JM, The Cyclin Cln1 controls polyploid titan cell formation following a stress-induced G2 arrest in cryptococcus. MBio 2021, 12:e0250921. 34634930 10.1128/mBio.02509-21 PMC8510536

[85] Cao C, Wang K, Wang Y, Liu T-B, Rivera A, Xue C: Ubiquitin proteolysis of a CDK-related kinase regulates titan cell formation and virulence in the fungal pathogen *Cryptococcus neoformans*. Nat Commun 2022, 13:6397. 36302775 10.1038/s41467-022-34151-6 PMC9613880

[86] Liu K-H, Shen W-C: Sexual differentiation is coordinately regulated by *Cryptococcus neoformans* CRK1 and GAT1. Genes 2020, 11:669, 10.3390/genes11060669 32575488 PMC7349709

[87] Wang J-M, Zhou Q, Cai H-R, Zhuang Y, Zhang Y-F, Xin X-Y, Clinicopathological features of pulmonary cryptococcosis with cryptococcal titan cells: a comparative analysis of 27 cases. Int J Clin Exp Pathol 2014, 7:4837–4846. 25197354 PMC4152044

[88] Subedi N, Bhattarai S, Ranabhat S, Sharma BK, Baral MP, Upadhyaya TL: Disseminated cryptococcosis in a deceased with HIV-1 diagnosed by minimally invasive tissue sampling technique. Clin Case Rep 2021, 9:1667–1671. 33768911 10.1002/ccr3.3865 PMC7981628

[89] Freitas GJC, Gouveia-Eufrasio L, Emidio ECP, Carneiro HCS, de Matos Baltazar L, Costa MC, The dynamics of *Cryptococcus neoformans* cell and transcriptional remodeling during infection. Cells 2022, 11:3896, 10.3390/cells11233896 36497155 PMC9740611

[90] Mozo EG, Ross O, Yuecel R, Dambuza IM, Mukaremera L: Human Plasma-Like Medium (HPLM) induces *Cryptococcus neoformans* in vivo cell morphologies. mSphere. 2024, 9:e0028124. 38771036 10.1128/msphere.00281-24 PMC11332328

[91.••] Alanio A, Vernel-Pauillac F, Sturny-Leclère A, Dromer F: *Cryptococcus neoformans* host adaptation: toward biological evidence of dormancy. MBio 2015, 6:e02580–14, 10.1128/mBio.02580-14. 25827423 PMC4453510

[92] Jung S-H, Ryu C-M, Kim J-S: Bacterial persistence: fundamentals and clinical importance. J Microbiol 2019, 57:829–835. 31463787 10.1007/s12275-019-9218-0

[93] de Castro RJA, Rêgo MTAM, Brandão FS, Pérez ALA, De Marco JL, Poças-Fonseca MJ, Engineered fluorescent strains of *Cryptococcus neoformans*: a versatile toolbox for studies of host-pathogen interactions and fungal biology, including the viable but nonculturable state. Microbiol Spectr 2022, 10:e0150422. 36005449 10.1128/spectrum.01504-22 PMC9603711

[94] Day AM, Quinn J: Stress-activated protein kinases in human fungal pathogens. Front Cell Infect Microbiol 2019, 9:261. 31380304 10.3389/fcimb.2019.00261 PMC6652806

[95.••] Ke W, Xie Y, Chen Y, Ding H, Ye L, Qiu H, Fungicide-tolerant persister formation during cryptococcal pulmonary infection. Cell Host Microbe 2024, 10.1016/j.chom.2023.12.012 276–289.e7. 38215741

[96] Lin J, Pham T, Hipsher K, Glueck N, Fan Y, Lin X: Immunoprotection against Cryptococcosis offered by Znf2 depends on capsule and the hyphal morphology. MBio 2022, 13:e0278521. 35012334 10.1128/mbio.02785-21 PMC8749420

